# Autochthonous transmission of Chagas disease in Rio de Janeiro State, Brazil: a clinical and eco-epidemiological study

**DOI:** 10.1186/s12879-014-0732-8

**Published:** 2015-01-08

**Authors:** Luiz Henrique Conde Sangenis, Roberto Magalhães Saraiva, Ingebourg Georg, Liane de Castro, Valdirene dos Santos Lima, André Luiz R Roque, Samanta Cristina das Chagas Xavier, Laura Cristina Santos, Fabiano A Fernandes, Otília Sarquis, Marli Maria Lima, Filipe Aníbal Carvalho-Costa, Márcio Neves Bóia

**Affiliations:** Laboratório de Pesquisa Clínica em Doença de Chagas, Instituto Nacional de Infectologia Evandro Chagas, Fundação Oswaldo Cruz, Rio de Janeiro, RJ Brazil; Laboratório de Imunodiagnóstico, Instituto Nacional de Infectologia Evandro Chagas, Fundação Oswaldo Cruz, Rio de Janeiro, Brazil; Laboratório de Farmacocinética, Instituto Nacional de Infectologia Evandro Chagas, Fundação Oswaldo Cruz, Rio de Janeiro, Brazil; Laboratório de Biologia de Tripanossomatídeos, Instituto Oswaldo Cruz, Fundação Oswaldo Cruz, Rio de Janeiro, Brazil; Laboratório de Doenças Parasitárias, Instituto Oswaldo Cruz, Fundação Oswaldo Cruz, Rio de Janeiro, Brazil; Laboratório de Ecoepidemiologia da Doença de Chagas, Instituto Oswaldo Cruz, Fundação Oswaldo Cruz, Rio de Janeiro, Brazil; Laboratório de Sistemática e Bioquímica, Instituto Oswaldo Cruz, Fundação Oswaldo Cruz, Rio de Janeiro, Brazil; Laboratório de Biologia e Parasitologia de Mamíferos Silvestres Reservatórios, Instituto Oswaldo Cruz, Fundação Oswaldo Cruz, Rio de Janeiro, Brazil

**Keywords:** Chagas disease, Trypanosoma cruzi, Epidemiology, Clinical, Transmission, Genotypes, Triatoma vitticeps, Rio de Janeiro

## Abstract

**Background:**

After the control of the main modes of Chagas disease (CD) transmission in most endemic countries, it is important to identify the participation of native sylvatic vectors in CD transmission. Although CD is not considered endemic in Rio de Janeiro State (RJ), Brazil, we identified patients with CD born in RJ and investigated the possible autochthonous transmission in the state.

**Methods:**

Patients born in RJ and followed in our institution between 1986 and 2011 were retrospectively analyzed. The cases identified as autochthonous transmission were submitted to epidemiological, clinical, serological, parasitological and molecular studies. Sectional field study with serological survey, research of sylvatic reservoirs and vectors was conducted in rural areas where patients were born.

**Results:**

Among 1963 patients, 69 (3.5%) were born in RJ. From these, 15 (21.7%) were considered to have acquired the infection by autochthonous transmission. Cardiac form was the commonest form of presentation (60%). In rural areas in RJ northern region, sylvatic cycles of *Trypanosoma cruzi* and domestic invasion by *Triatoma vitticeps* were identified, and CD prevalence among inhabitants was 0.74%.TcI genotype was identified in sylvatic reservoirs and vectors. The genotype (mixed infection TcI/TcVI) could be identified in one of the autochthonous cases.

**Conclusions:**

The autochthonous vectorial transmission of CD occurs in RJ, probably due to wild cycles of *T. cruzi* and sylvatic vectors, such as *T. vitticeps*. Therefore, the health authorities should evaluate if RJ should be included in the original endemic area of CD and CD should be included in the diagnostic work out of cardiomyopathy of patients born in RJ. Moreover, control and educational measures should be put into place in the risk areas.

## Background

Although the main modes of Chagas disease (CD) transmission (domestic vectorial transmission by *Triatoma infestans* and *Rhodnius prolixus,* and blood transfusion) are controlled in most endemic countries, 28 million people are still at risk to acquire CD in Latin America [1]. Vectorial transmission by native vectors maintains the risk of CD transmission in Brazil and other areas of the Americas [2-7]. In Brazil, the original endemic area was based on the domestic vector distribution, primarily in the regions where *T. infestans* was present as well as that of native triatomine bugs adapted to the domestic environment, such as *Panstrongylus megistus* and *Triatoma brasiliensis* [8,9]. Therefore, CD was never considered endemic in Rio de Janeiro State (RJ), given the apparent domestic distribution of triatomine bugs and vectorial transmission [8-10].

Most patients with CD diagnosed in RJ over past decades were imported from endemic areas as metropolitan regions in southeastern Brazil were the main destinations of migration flows from rural regions during the 20^th^ century [11-14]. However, cases possibly acquired in RJ were identified and mostly attributed to blood transfusion, travel history of patients born in RJ to other endemic states, and rarely to congenital transmission [12]. Nevertheless, the transmission route in patients with CD born in RJ remained unclear in some cases that could be autochthonous cases [11,12,15].

The possible occurrence of autochthonous cases of CD in RJ is studied since 1943 when sylvatic reservoirs and vector infected by *T. cruzi* were identified in Rio de Janeiro city [16]. Some studies documented serologically positive cases of the disease but did not investigate other possible routes of transmission [11,13,15,17]. Despite the occurrence of domestic invasion by *Triatoma vitticeps* at various locations in the state of RJ and its recognized role as CD transmitter in Espírito Santo State (ES), the long interval between feeding and defecation has always put their role in CD transmission into question [18-25]. Therefore, CD autochthonous cases in RJ were not fully recognized. However, over the past few years, focus of CD had occurred in rural areas of RJ where specimens of *T. vitticeps* were collected inside houses [22,23]. Human cases of CD were identified in these locations, calling attention to the possible vectorial transmission of the disease in this state.

The present study describes the clinical and epidemiological characteristics of 15 patients with CD acquired in RJ and through a cross-sectional field study in rural areas of the state, gives insight into the mechanism of vectorial transmission in this state.

## Methods

### Study design and collection of epidemiological, clinical, and laboratory data

We retrospectively reviewed all patients with CD admitted and followed at the Instituto Nacional de Infectologia Evandro Chagas (INI), Oswaldo Cruz Foundation (FIOCRUZ), between 1986 and 2011. The mechanism of transmission was studied in all patients who were born in RJ and was based on the following criteria: (i) Congenital transmission: those who had mothers native to endemic regions of CD or who were serologically positive for CD without further epidemiological evidence for the risk of acquiring the disease; (ii) Transmission through blood transfusion: all patients with a history of transfusion of hemoderivatives or organ transplantations before 1992 were placed in this group, even if they showed other risk factors, such as having a mother from an endemic region or who lived in a rural area; (iii) Transmission outside RJ: those who, despite being born in RJ, had traveled or lived in known endemic areas in other states; (iv) Autochthonous transmission: all risk factors for acquiring CD were excluded (mothers from known endemic regions of CD, transfusion history of hemoderivatives or organ transplantation, living and traveling to endemic regions of CD outside RJ). Only the patients from the autochthonous group were included in this study.

The clinical classification followed the Brazilian Consensus on Chagas Disease: indeterminate form, cardiac form (stage A, B1, B2, C, and D), digestive form (megaesophagus grade I, II, III, and IV, as well as megacolon), and mixed form (cardiodigestive) [10].

The following variables were analyzed in the patients with signs of autochthonous transmission: age; sex; serological, parasitological, molecular, radiological, electrocardiographic (ECG), and echocardiographic findings; disease progression; death; treatment; clinical form; possible infection site; residence; eating habits; and knowledge of vectors. Epidemiological data were obtained from medical records and patient interviews and included exposure to vectors; blood transfusions; organ transplants; maternal history of CD; residence in rural areas; travel history from RJ to endemic regions; and consumption of risk food for the transmission of *T. cruzi*, such as meat of hunted wild mammals and beverages produced by artisans (e.g., juice from sugarcane and açaí palm (*Euterpe edulis*) native to the Brazilian Atlantic Forest). Death certificates of patients’ mothers were also investigated, and the maternal serology for CD was tested whenever possible.

A field study was carried out in rural areas of north of RJ where the most recent cases of CD identified in this study occurred with evidence of vectorial transmission. Farms were visited, and blood samples were collected from residents for serological testing for CD. Small, wild, synanthropic mammals were captured, and the prevalence of *T. cruzi* infection was studied by parasitological and serological methods. Triatomine bugs found in houses were collected and their feces were searched for *T. cruzi* infection by direct microscopic examination and polymerase chain reaction (PCR).

### Case-series study

#### Serology

Two serological techniques were used in the diagnosis of CD (indirect immunofluorescence [IIF; WAMA Diagnóstica, São Paulo, Brazil] and enzyme-linked immunosorbent assay [ELISA; Biozima Chagas kit, Buenos Aires, Argentina]) to detect anti-*T. cruzi* antibodies in the serum of the same sample obtained via peripheral venipuncture [10].

#### Chest radiograph with esophageal contrast, electrocardiography, echocardiography, and digestive endoscopy

All patients underwent posterior-anterior and latero-lateral chest radiographs with contrast esophagogram and 12-lead ECG. All patients with Chagas cardiomyopathy underwent 2-dimensional echocardiography. The ejection fraction was calculated using the Teicholz index [26]. Patients with constipation for more than 7 days, dysphagia, or positive radiological findings of megaesophagus underwent digestive endoscopy.

#### Indirect xenodiagnoses

Peripheral blood samples (8 mL) were collected via venipuncture into vials containing heparin from patients with autochthonous evidence of vectorial transmission and were sent to the Laboratory of Parasitic Diseases, Instituto Oswaldo Cruz (IOC)/FIOCRUZ. Each sample was placed in a glass vial covered with latex film and stored at 37°C. Forty third-stage and fourth-stage nymphs of *T. infestans* and *T. vitticeps* were placed in another glass container covered with fenestrated tissue and fed blood. After 45 days, each nymph was analyzed individually. The intestines were dissected, macerated, diluted in 0.9% saline and analyzed with a light microscope (400x) for *T. cruzi*.

#### Hemoculture for T. cruzi

A 5-mL peripheral blood sample was collected from each autochthonous patient into a vial containing ethylenediaminetetraacetic acid (EDTA) and immediately sent to the Laboratory of Tripanosomatid Biology at IOC/FIOCRUZ. The sample was grown on liver infusion tryptose medium (LIT) for hemoculture and monitoring, as described by Lisboa et al. [27].

#### Polymerase chain reaction

Two 5-mL samples of peripheral blood were collected from each autochthonous patient into vials containing EDTA. One of them was sent to the Pharmacokinetic Laboratory at INI/FIOCRUZ for amplification of kinetoplast DNA (kDNA) by performing PCR, using total blood and commercial kits (QIAGEN® [DNA Blood Mini Kit]), as described previously [28,29]. The other sample was sent to the Laboratory of Tripanosomatid Biology at IOC/FIOCRUZ for mini-exon multiplex PCR and restriction fragment length polymorphism (RFLP) analysis of the gene histone H3 and gp72 to identify the *T. cruzi* genotypes (TcI-TcVI) in serum, as described above, and according to the current *T. cruzi* nomenclature [30-33].

### Field study

#### Study areas

**Figure 1:**
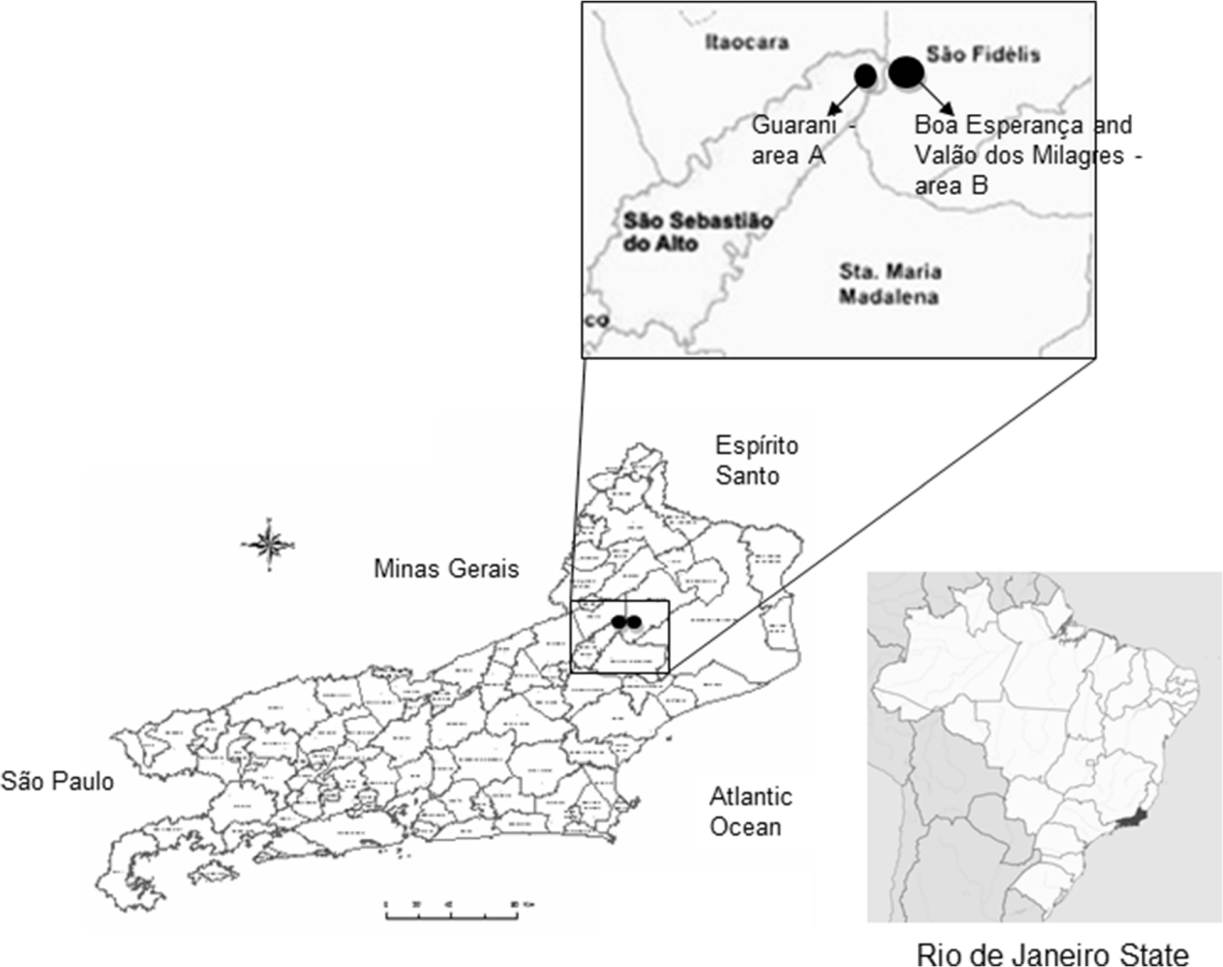
**Study areas.** Guarani, municipality of São Sebastião do Alto, and Boa Esperança and Valão dos Milagres, municipality of São Fidélis, located to the north of Rio de Janeiro State.

Two rural sites north of RJ were studied. Area A included the Guarani of Ipituna, district of Valão do Barro, municipality of São Sebastião do Alto. Area B included the towns of Boa Esperança and Valão dos Milagres, district of Cambiasca, municipality of São Fidélis (Figure 1). The study areas met all the following criteria: (i) recently observed autochthonous cases of CD identified in this study, (ii) controlled environmental surveillance reporting cases of regular collection of triatomine bugs from houses, (iii) the presence of Program of Community Health or Family Health Strategy workers, and (iv) municipalities that had higher number of autochthonous cases of CD observed in the present study.

The study area is located 250 km from the state capital (Rio de Janeiro municipality) in a transition area between the Serrana and Northern regions at an altitude of approximately 200 m (21°45′18″S 41°58′12″W [area A] and 21°43′23″S 41°52′28″W [area B]). The climate is tropical with 1000–1500 mm annual rainfall. The region has many valleys that are crossed by the Negro and Grande rivers; these rivers join Cambiasca to form Dois Rios river, one of the main tributaries of Paraiba do Sul river. The landscape is degraded with many pastures and secondary forest niches with fragments of Atlantic Forest in the tops of the hills. Most houses in the study area are small rural properties of agricultural families. Vegetables and dairy farming are the main economic activities of the region.

#### Field study participants

All houses in the region were visited by Family Health Strategy workers, and residents in 71.1% of them (n = 106 and 139 in areas A and B, respectively) filled out the questionnaire and donated a blood sample. A total of 404 peripheral blood samples were collected on filter paper (Klabin n^o^ 80) via finger puncture, packed in individual plastic bags, and sent to the immunodiagnostic laboratory at INI, where the filters were eluted in distilled water and the serological reaction was carried out by performing IIF and ELISA. The age of participants ranged from 2 to 90 years old. Epidemiological information, including housing characteristics; the presence of pets; and the consumption of wild animals, sugarcane juice, and açaí palm juice, were recorded on the questionnaires. Participant knowledge about triatomine bugs was determined using a display case containing 2 adult specimens and 2 nymphs of *T. vitticeps* and *T. infestans* species.

#### Small mammal trapping and sample collection

Tomahawk (Tomahawk®) and Sherman (Sherman®) live traps were placed for 4 consecutive nights in several sites around the houses in the study areas to capture small, wild, synanthropic mammals between July 12^th^ and 15^th^, 2010. Forty-five traps of each model baited with a mixture of peanut butter, banana, oat, and bacon were used per night, totaling 360 traps. The identification of mammals was made by morphological characteristics and karyriologic analyses as previously described [34]. In a field laboratory set up exclusively for this purpose, blood samples were collected via intracardiac puncture and processed as follows: (i) 0.6 mL of blood was cultured in two tubes containing Novy-Mc Neal-Nicoly medium (NNN) with a LIT overlay (hemoculture); and (ii) the remaining blood was centrifuged and the serum was stored at −20°C prior to serological analysis using IIF, as previously described [35]. The *T. cruzi* isolated obtained was characterized using mini-exon multiplex PCR, as described by Fernandes et al. [30].

#### Triatomine bugs

Between July 2010 and November 2012, triatomine bugs were manually collected with forceps from houses in the study areas and placed into individual plastic vials with a screw cap on which the location and collection date were written. Triatomine bugs collected in the same period in other localities of the municipalities where the field study was developed were also analyzed. Four different researchers captured the triatomines: two in the area A and two in the area B. However, around 30% of the triatomines were collected by the residents themselves and given to the researchers or community health workers. No dislodging agent was used to collect triatomines. Most of the captures were done during the day and flashlights were used whenever the sunlight was insufficient. The samples were sent to the Eco-epidemiology Laboratory of Chagas Disease at IOC/FIOCRUZ where light microscopy (400×) was used to identify tripanosomatids in the feces. The bugs that arrived alive in the laboratory were selected for mini-exon multiplex PCR study of the intestines for *T. cruzi* infection, according to previously described methodology [30,36]. Beyond the proportion of natural infection by *T. cruzi,* others two entomological indicators (density index and crowding index) from the two study areas were calculated based on the number of triatomine bugs collected and houses visited by the study team, as recommended by WHO and discussed by Dias and Diotaiuti [37].

#### Ethical considerations

This study was approved by the Human Research Ethics Committee at INI/FIOCRUZ (license 016/2011). All patients and field study participants signed informed consents, indicating agreement to participate in the study. The study of wild animals was based on protocols that were approved by the FIOCRUZ Committees of Bioethics (license 0015–07), and wild animal captures were licensed by the Brazilian Institute of Environment and Renewable Natural Resources (IBAMA/CGFAU/LIC) (license 3665–1).

## Results

### Case-series study

**Figure 2:**
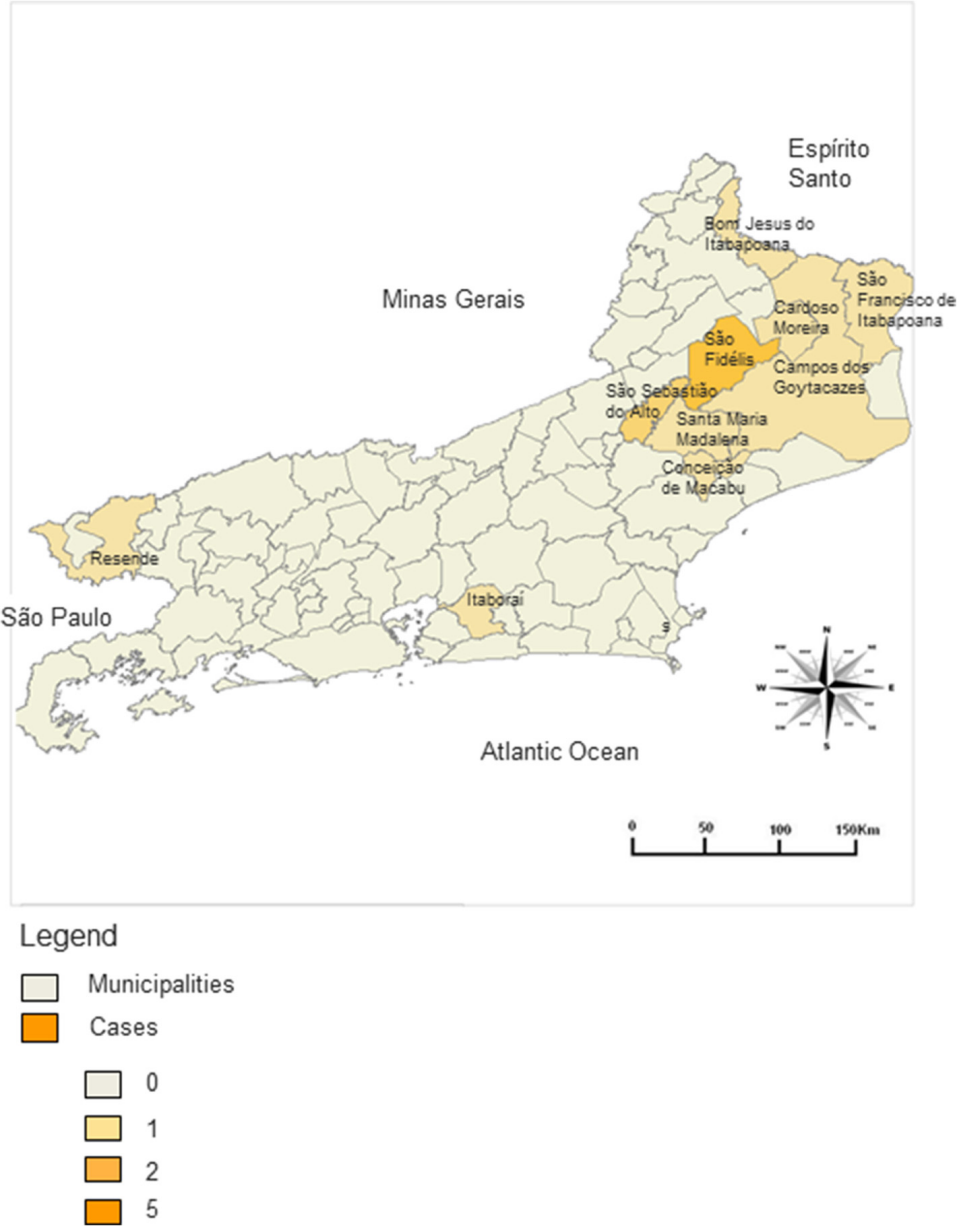
**Distribution of 15 cases of native vectorial transmission of Chagas disease according to city of birth in Rio de Janeiro State.**

From a total of 1963 patients, 69 (3.5%) were native to RJ. The mechanism of transmission was considered to be congenital in 32 (46.4% of the cases), blood transfusion in 10 (14.5%), transmission outside RJ in 7 (10.1%), and autochthonous in 15 patients (21.7%). The patients from the first three subgroups were all born in urban areas. Most of the 15 patients classified as autochthonous vector transmission were natives from rural areas of the state, with 13 originating from municipalities in the northern areas of the RJ: São Fidélis (n = 5), São Sebastião do Alto (n = 2), Campos dos Goytacazes (n = 1), Santa Maria Madalena (n = 1), Conceição de Macabu (n = 1), Bom Jesus do Itabapoana (n = 1), Cardoso Moreira (n = 1), and São Francisco de Itabapoana (n = 1). One patient originated from Itaboraí (metropolitan region) and another from Resende (southern RJ region) (Figure 2). There was insufficient epidemiological information for 5 patients to define the form and site of transmission.

All following data refer to the patients considered to have acquired CD by autochthonous vectorial transmission. Most patients were male (12 male [80%] and 3 female [20%]). All patients stated that they had resided in wattle and daub houses for the first decades of their life and 7 had seen triatomine bugs in their houses. All patients consumed bush meat (armadillo, opossum, cavy, and pacas) and sugarcane juice. Only 2 patients mentioned sporadic consumption of açaí palm juice.

From the 15 cases, 5 were considered to present the indeterminate form, 8 the cardiac form, 1 the digestive form and 1 the mixed cardiac and digestive form. From the 8 patients with the cardiac form, five presented the stage A, two the stage B and one the stage C of the cardiac form. The patient with the digestive form presented megaesophagus grade III and the one with the mixed form presented megaesophagus grade II (Table 1). Two young patients with the indeterminate form were treated with benznidazole. Of the 8 patients with CD cardiac form, 3 patients died before the start of the study: 1 due to sudden death, 1 due to refractory heart failure, and 1 due cervical cancer.

**Table 1:** **Clinical data of patients Chagas disease (CD), from Rio de Janeiro with autochthonous vectorial transmission**

**Case**	**Clinical and laboratory data**										
	**Age**	**Clinical form**	**Follow-up**	**IIF**	**ELISA**	**ECG**	**ECO (EF%)**	**Chest x-ray**	**Xeno**	**kDNA PCR**	**m.exon PCR**
1	34	CF	Death	1/320	1/640	PR, PVC	Hypokinesia (45)	Normal	Neg	ND	ND
2	40	CF	Death	1/640	1/1280	RBBB, LAHB, PVC, PAC	Hypokinesia ↑ LA (35)	Cardiomegaly	ND	ND	ND
3	48	CF	Alive	1/1280	RI 4.8	RBBB, LAHB	Normal (75)	Normal	Neg	Neg	Neg
4	65	CF	Death	1/320	1/640	RBBB, LAHB, PVC	Delayed relaxation (60)	Normal	ND	ND	ND
5	49	IF	Alive	1/320	RI 6.6	Normal	PDA (62)	Normal	Neg	Neg	Neg
6	46	CF	Alive	1/640	RI 4.0	RBBB, LAHB	Normal (66)	Normal	Neg	Neg	Neg
7	55	CF	Alive	1/640	RI 5.0	LBBB, PVC	Normal (75)	Normal	Neg	Pos	Neg
8	57	CF/DF	Alive	1/640	RI 4.4	RBBB	Normal (76)	Megaesophagus	Pos	Neg	Neg
9	33	CF	Alive	1/320	RI 5.7	Primary ST-T changes	Hypokinesia ↑ VE (53)	Normal	Neg	Neg	Neg
10	41	IF	Alive	1/40	RI 2.0	Normal	Normal (68)	Normal	Neg	Neg	Neg
11	50	DF	Lost to follow- up	1/40	RI 1.5	Normal	--	Megaesophagus	ND	ND	ND
12	24	IF	Alive	1/80	RI 4.1	Normal	Normal (65)	Normal	Neg	Pos	Pos
13	15	IF	Alive	1/160	RI 6.2	Normal	Normal (67)	Normal	Neg	Pos	Neg
14	34	IF	Alive	1/160	RI 6.4	Normal	Normal (68)	Normal	Neg	Pos	Neg
15	68	CF	Alive	1/80	RI 1.1	RBBB	Delayed relaxation (74)	Cardiomegaly	Neg	Neg	Neg

CF = cardiac form, DF = digestive form, ECG = electrocardiography; ECO = echocardiography, EF = ejection fraction, ELISA = enzyme-linked immunosorbent assay, IF = indeterminate form, IIF = indirect immunofluorescence, kDNA = kinetoplast DNA, LA = left atrium, LAHB = left anterior hemiblock, LBBB = left bundle branch block, LV = left ventricle, m.exon = mini-exon multiplex, ND = not determined; PAC = premature atrial complex, PCR = polymerase chain reaction, PDA = patent ductus arteriosus, PVC = premature ventricular complex, PR = pacemaker rhythm, x –ray = radiograph, RBBB = right bundle branch block, RI = reactivity index.

The ages described are from the beginning of the patients’ follow-up at our outpatient service. Except for cases 1, 2 and 4 who died before the start of this study, all clinical and laboratory data were reevaluated to check for serological and molecular diagnosis and the clinical form presentation. All patients presented negative hemoculture for *T. cruzi*.

**Figure 3:**
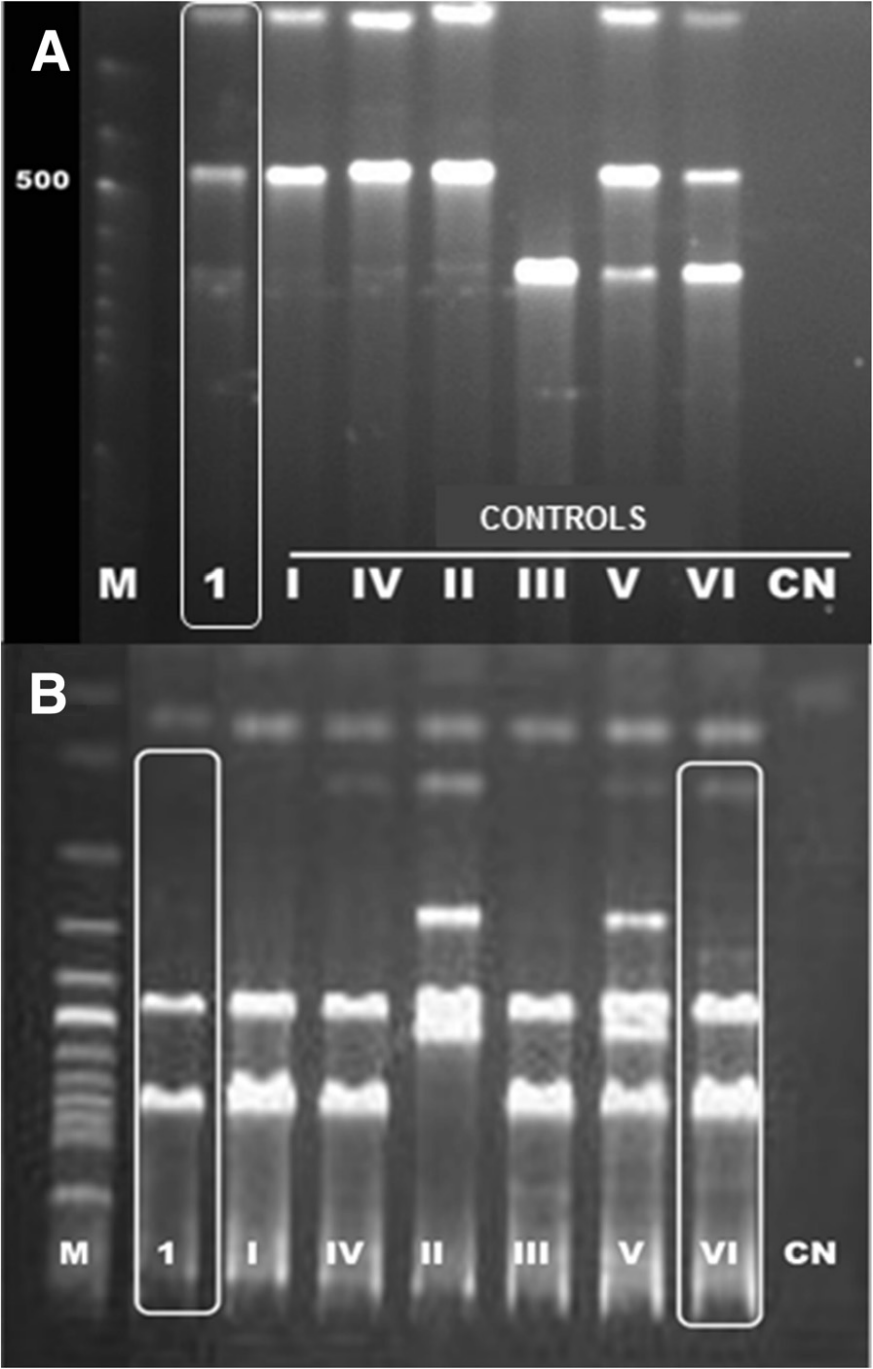
**Agarose gel electrophoresis with the molecular characterization of mixed infection with genotypes DTUs TcI + TcII / TcV / TcVI** ***T. cruzi*** **by RFLP PCR. A.** Profile fragments of the PCR products H3 gene after digestion with restriction enzyme AluI; 1- patient (case 12), positive controls TcI-VI, CN-negative reaction control. **B.** Profile fragments of the PCR products of the gp72 gene after digestion with restriction enzyme TaqI; 1- patient (case 12), positive controls TcI-VI, CN-negative reaction control. M-molecular weight 100 bp.

The 15 patients considered to have acquired CD by autochthonous vectorial transmission presented positive IIF and ELISA CD serological tests. Of these, 12 underwent indirect xenodiagnoses, with 1 positive result, and 11 underwent molecular studies. Four patients presented positive result in kDNA PCR and one patient presented a mixed infection with TcI and TcII genotypes in the mini-exon multiplex PCR and the DTUs were characterized as TcI and TcVI by performing RFLP analysis of the gene histone H3 and gp72 (Figure 3). Hemoculture for *T. cruzi* was negative in all samples.

### Field study

In the field study, 245 rural properties in Northern region of RJ were studied, 84% of which had brick walls, 16% had wattle and daub walls, and 84% had tile roofs without ceilings. Knowledge of the vector was reported by 35% of residents. From the 404 blood samples collected from participants, 3 were found be serologically positive for CD (1 adolescent and 2 adults), yielding a prevalence of 0.74%. The 3 positive samples belonged to patients previously identified with CD and followed at the outpatient center of the INI/FIOCRUZ and who also resided in the study areas (see Table 1, cases 10, 13, and 14).

A total of 34 small wild mammals were captured including two marsupial species (*Didelphis aurita* and *Philander frenatus*) and four rodent species (*Akodon cursor*, *Nectomys squamipes*, *Oligoryzomys nigripes* and *Rattus rattus*). *T. cruzi* infection was found in 3 specimens (8.8%); two by serological assay (*D. aurita* and *R. rattus*) and one by positive hemoculture (*A. cursor*). The positive hemoculture was characterized as TcI (Table 2) and deposited in the *Trypanosoma* from Sylvatic and Domestic Mammals and Vector Collection of Oswaldo Cruz Foundation (ColTryp 00356).

**Table 2:** **Serology, hemoculture and PCR for** ***Trypanosoma cruzi*** **of small mammals captured to the north of RJ**

**Species**	**Captured**	**Positive IIF**	**Positive hemoculture**	**Genotype mini-exon PCR**	**% positive**
*Didelphis aurita*	3	1	-	-	33.3
*Philander frenatus*	2	-	-	-	-
*Akodon cursor*	24	-	1	TcI	4.2
*Nectomys squamipes*	1	-	-	-	-
*Oligoryzomys nigripes*	2	-	-	-	-
*Rattus rattus*	2	1	-	-	50
Total	34	2	1		8.8

IIF = indirect immunofluorescence, PCR = polymerase chain reaction, RJ = Rio de Janeiro State.

A total of 49 triatomine bugs of the species *T. vitticeps* were collected from inside houses (42 adults and 7 nymphs). In the two study areas, the study team collected 25 triatomine bugs in 20 (8.2%) of the 245 homes visited while up to 35% of the residents who answered the questionnaires reported that they had already seen triatomine bugs inside their houses. The density index was 0.102 and the crowding index 1.25.Three out of 26 triatomine bugs (11.5%) whose feces were analyzed under the light microscope showed tripanosomatids similar to *T. cruzi*. Other triatomine bugs could not be analyzed because they were very dry and without any intestinal contents. TcI genotype was identified in 8 out of 11 (73%) triatomine bugs analyzed by mini-exon multiplex PCR (Table 3).

**Table 3:** **Data from** ***Triatoma vitticeps*** **bugs collected indoors in 3 municipalities to the north of RJ**

			**Number of triatomine bugs**									
**Location**	**District**	**Municipality**	**Adults**	**Nymphs**	**Male**	**Female**	**Microscopy**	**Positive**	**%**	**PCR**	**Positive**	**%**
Guarani	Valão do Barro	São Sebastião do Alto	8	--	3	5	5	--	--	4	3	75
Cabeceira	Valão do Barro	São Sebastião do Alto	2	--	--	2	1	--	--	1	--	--
Centro	São Sebastião do Alto	São Sebastião do Alto	6	--	2	4	3	--	--	2	2	100
Centro	Valão do Barro	São Sebastião do Alto	4	--	1	3	1	--	--	1	1	100
Valão dos Milagres	Cambiasca	São Fidélis	3	--	1	2	2	1	50	--	--	--
Boa Esperança	Cambiasca	São Fidélis	13	--	2	6	4	--	--	--	--	--
São Tomé	Cambiasca	São Fidélis	1	--	--	1	-	--	--	--	--	--
Grumarim	São Fidélis	São Fidélis	1	--	--	1	1	1	100	--	--	--
Retiro Saudoso	Colônia	São Fidélis	1	6	--	1	7	1	14.3	--	--	--
Colégio de Cima	Colônia	São Fidélis	--	1	--	-	-	--	--	--	--	--
Triunfo	Triunfo	Santa Maria Madalena	3	--	1	2	2	--	--	3	2	66
Total			42	7	11	31	26	3	11.5	11	8	73

PCR = polymerase chain reaction, RJ = Rio de Janeiro State.

## Discussion

In this paper, we were able to identify cases of autochthonous CD transmission within a population of patients born in RJ. Moreover, among patients with CD born in RJ followed at our institution, congenital transmission was the most common mode of CD transmission, while autochthonous transmission was the second most common mode and transfusion was the third most common mode of CD transmission. This is a clear contrast with the previous concept that transfusion would be the main mode of CD transmission in Brazilian urban areas [38,39]. In fact, a comprehensive study carried out in 1965 found only 3 cases of transfusional transmission in patients born in RJ [12]. Transfusional transmission in Brazil may have been overestimated, considering that a large proportion of donated blood in cities had been serologically screened, even without strict governmental control [38]. Together with the fact that congenital transmission is more common in other South American countries, such as Bolivia, Argentina, and Chile, new studies on the epidemiological nature of CD in urban areas of Brazil are necessary [40-42]. Most of the patients with a history compatible with autochthonous vectorial transmission came from a common geographic rural area of RJ, comprising the serrana, north and northwest regions up to the state boundary with ES, where we found *T. vitticeps* specimens inside houses. The visits conducted by environmental surveillance agents in the present study revealed that triatomine bugs are regularly found in houses in at least 5 municipalities of the region (São Fidélis, São Sebastião do Alto, Santa Maria Madalena, Conceição de Macabu, and Trajano de Moraes). Other studies also described the presence of *T. vitticeps* specimens in houses in the same region [18-21,23]. Furthermore, other studies also described patients with CD born in the Northern region of RJ [11,15].

The autochthonous CD transmission in the Northern region of RJ probably occurs by sporadic invasion of homes by *T. vitticeps* which are infected by *T. cruzi* due to a sylvatic cycle including sylvatic reservoirs living in areas surrounding the houses. This is similar to the CD epidemiological behavior reported in ES, which is usually associated with the presence of *T. vitticeps* in homes [24,43-45]. On the other hand, the prevalence of CD in ES and in the Northern region of RJ described by us is low, as *T. vitticeps* seldom colonizes houses and due to its low vector potential [21,24,25,44]. In fact, nymphs were captured in only 5% of the houses where triatomine bugs were found in this study, which demonstrates that this species do not usually adapt to the domestic environment, unlike other wild vectors that have adapted to domestic environments, such as *P. megistus*, *Triatoma sordida*, and *T. brasiliensis* [46]. Despite the low entomological indicators, the triatomines collected in this study presented high level of natural infection by *T. cruzi,* as showed in others researches, which may contribute to the risk of CD transmission [21,47]. Home invasion by sylvatic vectors is a great challenge for epidemiological surveillance in several countries. Enzootic cycles of *T. cruzi* involved in the emergence of human cases of CD have been documented in other regions of Brazil, Latin America and in the USA [2-7,48].

Many factors may influence the presence of autochthonous cases of CD in RJ, such as past and present occurrence of *T. vitticeps*, a species endemic in the Northern region of RJ; continuous deforestation with successive economic cycles in the region; the presence of rural inhabitants with permanent farming settlements; and a high number of dwellings of low socioeconomic status [19-21,49]. Additionally, vectors which usually invade but do not colonize homes are attracted to house lights and the absence of ceilings, window glass, and screens in many of the houses visited in the field study provides the best conditions for invasion [21,23]. The consume of wild animals may also contribute to the emergence of human cases of CD not only due to the risk of accidental infection from undercooked meat, but also due to the decrease in wild food sources for triatomine bugs [46,50-52]. Other factor that might contribute to the occurrence of autochthonous cases of CD in RJ would be the consumption of beverages, such as sugarcane juice and açaí palm juice [52,53]. However, this is unlikely as sugarcane juice consumption was related to outbreaks of acute CD due to the ingestion of large numbers of parasites when triatomine bugs were crushed together with the plant, which was not observed in RJ [53,54]. Moreover, there is no previous description of CD transmission by consumption of the *E. edulis* palm juice, which is the açaí palm commonly found in the Atlantic Forest*.* Furthermore, only two patients reported the sporadic consumption of the *E. edulis* palm juice and this mode of CD transmission would be associated with acute CD cases occurring within families which are not described in RJ [52]. Another possible mechanism of oral transmission is the accidental contamination of food with feces of infected triatomine bugs or by *T. cruzi* in the anal gland contents of infected opossums [40,55,56]. The present study documents the natural infection of triatomine bugs and wild animals by *T. cruzi* in areas surrounding the houses in the field study sites, including 1 opossum (*D. aurita*), which supports this hypothesis. Therefore, the vector contaminative transmission is the most probable among the 15 patients but we cannot completely rule out other possibilities, such as the consumption of food contaminated with triatomine bugs feces or anal gland contents of opossums infected with *T. cruzi*.

Most of the *T. vitticeps* studied were collected during months when the temperature and relative humidity are usually the highest in Brazil, which coincides with the period when there is the greatest dispersion of triatomine bugs [21,57]. Additionally, more female bugs were captured, similar to the findings in other studies [21,47]. It was confirmed that the majority of triatomine bugs captured in houses did not have any intestinal contents, indicating the difficulty to find food sources in the wild, which could influence the dispersion intensity of the vectors [21].

TcI was found in all 3 participants of the protozoan life cycle (vector, animal reservoir, and human host). Moreover, in one human host was identified mixed infection by TcI and TcVI. Although the visceral forms (cardiac and digestive) of the disease predominated in autochthonous patients described in this study, it was not possible to correlate the severity of the presentation of the disease with the *T. cruzi* genotype as the specific genotype was identified in only one case. The low sensitivity of PCR in this study can be explained by the fact that all studied patients presented chronic CD and most of them moved away from the endemic areas decades ago. These conditions determine a low and irregular parasitemia and PCR performed in patients under such conditions usually do not present high sensitivity [58]. The low and irregular parisitemia may have also contributed to the discordance between indirect xenodiagnoses and PCR results as the samples used to run those exams were collected in different days. We consider that most of the cases described in this study were caused by TcI as this was the genotype identified from vectors and animal reservoirs. TcI is found in humans in the Amazon in Brazil and was also found in humans in the Northeast region of Brazil, and in Argentina and Bolivia [59-61]. The high prevalence of visceral forms found by us contradicts the idea that CD morbidity is lower in cases from RJ inhabitants [48]. Moreover, Chagas heart disease have already been documented in the Amazon, a region where TcI infections predominate [30,62,63]. TcVI was first identified in Brazil in vectors captured in Rio Grande do Sul State [33,59] and, thereafter, in patients affected by the outbreak of acute CD in Santa Catarina State in 2005 and in Minas Gerais State [64,65]. Study genotyping of *T. cruzi* in sylvatic cycles previously conducted in RJ had already revealed the complexity of genotypes in the state, with findings of TcI and TCII and mixed infection by mini-exon gene PCR [66]. Given the unprecedented findings regarding molecular epidemiology in RJ, documented in the present study, and the still poorly understood parasite-host interactions regarding the various lineages of *T. cruzi*, further studies are required to better clarify these correlations and their implications in the morbidity of CD not only in RJ but also in other regions of Brazil and Latin America.

With regard to the 2 autochthonous CD cases that did not originate from north of the state, we consider that the case from Resende (south of the state) was related to vectorial transmission by *T. infestans* and the mode of transmission of the case from Itaboraí (a metropolitan region) was similar to the other autochthonous cases described in this study. Several evidences indicate that the vector involved in the transmission to the patient from Resende was *T. infestans*. The patient was born in Engenheiro Passos, a rural district of Resende, in 1944 and *T. infestans* infected by *T. cruzi* was documented in the same area during the 1940s and 1950s [17,19]. *T. infestans* in this area probably originated from the municipality of Queluz, São Paulo State, which borders Engenheiro Passos [17,67]. The patient lived in a wattle and daub house and knew about triatomine bugs because they were spotted daily on the walls inside the house. Furthermore, in a study of 1952 carried out in South of RJ no other vector apart from *T. infestans* was documented and we did not find any publication referring to the presence of other triatomine bugs invading houses in Resende [17]. Regarding the case from Itaboraí, both oral and vectorial transmission are possible to have occurred as the patient frequently ate opossums hunted in the region and consumed sugarcane juice since childhood and he always lived in rural areas in the district of Sambaetiba in poor houses without ceilings or window screens. Moreover, *T. vitticeps* was found in rural areas of municipalities near Itaboraí [20,66]. However, in a separate study carried out in Sambaetiba, no relative had serological positive results for CD, and no vector was found in the houses or their annexes, even though the current address of the family is in the same district where the patient was born and lived the first years of life.

## Conclusions

The eventual vectorial transmission of CD occurs in RJ since many years ago, probably due to wild cycles of *T. cruzi* and sylvatic vectors, such as *T. vitticeps*. Therefore, even among patients born in RJ, CD should always be included in the diagnostic work out of cardiomyopathy. On the basis of the findings of this study, we recommend that the health authorities should evaluate if RJ should be included in the original endemic area of CD and that control and educational measures should be put into place in the risk areas.

## Abbreviations

CD: Chagas disease
DTUs: Discrete typing units
ECG: Electrocardiography
ECO: Echocardiography
EDTA: Ethylenediaminetetraacetic acid
ELISA: Enzyme-linked immunosorbent assay
ES: Espírito Santo State
FIOCRUZ: Oswaldo Cruz Foundation
IIF: Indirect immunofluorescence
INI: Instituto Nacional de Infectologia Evandro Chagas
IOC: Instituto Oswaldo Cruz
kDNA: Kinetoplast deoxyribonucleic acid
LIT: Liver infusion tryptose medium
NNN: Novy-Mc Neal-Nicoly medium
PCR: Polymerase chain reaction
RFLP: Restriction fragment length polymorphism
RJ: Rio de Janeiro State
WHO: World Health Organization
